# Signature genes associated with immunological non-responsiveness to anti-retroviral therapy in HIV-1 subtype-c infection

**DOI:** 10.1371/journal.pone.0234270

**Published:** 2020-06-24

**Authors:** Sukhvinder Singh, Jaideep S. Toor, Aman Sharma, Sunil K. Arora

**Affiliations:** 1 Department of Immunopathology, Postgraduate Institute of Medical Education & Research, Chandigarh, India; 2 Department of Internal Medicine, Postgraduate Institute of Medical Education & Research, Chandigarh, India; Emory University School of Medicine, UNITED STATES

## Abstract

**Objective:**

HIV-infected individuals undergoing therapy may show an immunological-discordant response to therapy, with poor CD4^+^ T cells recovery, despite viral suppression below the detection limit. The present study was carried out to delineate the underlying molecular mechanisms of immunological non-responsiveness to HIV therapy.

**Design:**

We conducted microarray-based whole gene expression profiles of 30 subjects infected with HIV-1 subtype C, in peripheral blood to discern the signature genes associated with immunological non-responsiveness. After a thorough analysis and comparison of gene-expression profiles, microarray data was validated via qRT-PCR approach.

**Results:**

Overall, we found 10 genes significantly up-regulated and 60 genes down-regulated (≥2-fold change) in immunological non-responders as compared to responders. Based on these results and pathway analysis of the protein-protein interaction, 20 genes were shortlisted for validation in human infected cases. We found statistically significant differences in expression levels of twelve genes *IL-1α*, *IL-1β*, *IL-7R*, *TNF-α*, *FoxP3*, *PDCD5*, *COX7B*, *SOCS1*, *SOCS3*, *RPL9*, *RPL23*, *and LRRN3* respectively among immunological non-responders compared to therapy responders, confirming their an intimate relationship with immunological responsiveness to therapy.

**Conclusions:**

Altogether, microarray and qRT-PCR validation results indicated that the aberrant expression of key genes involved in the regulation of T cell homeostasis, immune activation, inflammatory cytokine production, apoptosis, and immune-regulatory processes are possibly associated with immunological non-responsiveness in HIV-1 C infected individuals on ART.

## 1 Introduction

Infection with human immunodeficiency virus (HIV) is characterized by the progressive depletion of CD4^+^ T cells [1]. Disease progression in HIV-infected individuals can be delayed by treatment with highly active anti-retroviral therapy (HAART) [2]. The introduction of HAART has led to a decline in both mortality and morbidity due to HIV infection. The primary goal of HAART is to suppress the plasma viral load to an undetectable level, which normally should associate with an increase in the CD4^+^ T cell count in the majority of patients. However, 15–30% of treated patients show immunologic-virologic discordant responses to long-term HAART consisting of a lack of increase in the CD4^+^ T cell count despite significant suppression of HIV replication [3–6]. These individuals are referred to as ‘immunological non-responders’ (INRs) or ‘immunological failures’ (ART-IF). There is an overall increased risk for both AIDS and non-AIDS related complications, which can lead to enhanced morbidity and mortality in ART-IF [7, 8]. Clinical characteristics such as older age, nadir CD4^+^ T-cell count at the start of HAART, longer duration of HIV infection, and the existence of co-infections are known to be associated with poor CD4^+^ T cell count recovery [9–12]. Besides, the host genetic factors are also associated with heterogeneous CD4^+^ T cell recovery [13–15]. However, these factors do not provide a full explanation of the discordant response to therapy and hence, the mechanisms underlying the immunological non-responsiveness to HAART, are not well understood. Thus, there is a dire need to delineate the underlying fundamental mechanisms to identify the correlates of protection for better management of the HIV disease in these individuals.

In recent years, the advancements in gene expression analysis techniques especially microarray-based gene expression profiling have revolutionized the research, and have aided in the ability to elucidate the pathophysiology of infectious diseases, cancer, and treatment response [16–18]. In the present study, we conducted the microarray-based evaluation of gene expression profiles in whole peripheral blood of HIV-infected individuals undergoing HAART to characterize the signature genes associated with immunological non-responsiveness. The findings of this study indicate the association of key genes involved in the regulation of T cell homeostasis, immune activation, inflammatory cytokine production, apoptosis, and immune-regulatory processes with immunological non-responsiveness in HIV-1 subtype-C infected individuals on HAART.

## 2 Methodology

### 2.1 Ethical statement

The study was approved by the Institutional Ethics Committee (IEC) of the Postgraduate Institute of Medical Education & Research (PGIMER), Chandigarh, India vide agenda item No. NKG/926. An informed written consent was obtained from all the subjects involved in the study before obtaining a peripheral blood sample.

### 2.2 Study design

HIV-1 subtype-C infected individuals were recruited from the ART (Anti-retroviral treatment) clinic, Department of Internal Medicine and HIV ICTC (Intergraded Counselling and Testing Centre), Department of Immunopathology, PGIMER, Chandigarh, India. The study was carried out in **Immunological responders (ART-R; Group-I n = 10)**: included HIV-positive individuals who were on ART for at least one year or more and showed signs of response to the treatment in terms of gain of more than 150 cells/μL in CD4^+^ T cell count and current CD4 count >250 cells/μL); **Immunological failures (ART-IF; Group-II n = 10)**: included HIV-positive individuals who were on ART for at least one year or more showing undetectable viral load (<400 copies/mL) and CD4^+^ T cell count <250 cells/μL; **Treatment naïve (ART-N; Group-III n = 5)**: included HIV-positive ART naïve individuals with CD4^+^ T cell count >200 cells/μL, for comparison in order to evaluate appreciative changes in expression profile of certain genes on the institution of therapy and **Healthy control (HCs; Group-IV n = 5)**: included HIV-seronegative healthy individuals for control expression profile of genes. HIV patients co-infected with TB and other chronic co-infections (HCV, HBV) were excluded from the study.

### 2.3 Sample collection

Fresh peripheral blood (2.5 mL) was collected in Paxgene blood RNA tube (PreAnalytiX, Hombrechtikon, Switzerland) and incubated at room temperature for 2 hours or subsequently stored in -80°C till samples were processed for gene expression profiling. For other investigations, such as CD4 count, viral load and DNA extraction: 3.0 mL of peripheral blood was taken separately in a K3EDTA vacutainer tube (Becton Dickinson, USA).

### 2.4 RNA extraction

Total RNA was isolated from the peripheral blood using the Qiagen PaxGene RNA isolation kit (Hilden, Germany) as per manufacturer's guidelines. Assessment of RNA quality, integrity and purity was done through a Bio-analyzer 2100 (Agilent Technologies, Palo Alto, CA, USA). Samples with RNA integrity number (RIN) more than 8 were used for microarray gene expression profiling and analysis.

### 2.5 Microarray analysis

Affymetrix GeneChip^®^ PrimeView^™^ Human Gene Expression Array (Affymetrix, Santa Clara, California, USA) was used for gene expression profiling in the present study. Amplification, labeling, hybridization onto the GeneChip^®^ PrimeView^™^ Human Gene Expression Array, washing, and scanning (Affymetrix Gene Chip 3000 Scanner) were performed as per the manufacturer’s protocol.

### 2.6 Microarray data analysis

All scanned array images were visually inspected at Affymetrix AGCC portal. After the acquisition of microarray experiments images, raw datasets were extracted from each Cel file (raw intensity file). These raw datasets were separately analyzed using the GeneSpring GX12.1 software. All the microarray raw data (cel files) were pre-processed using the RMA (Robust Multichip Average) algorithm which consisted of three steps: background adjustment, normalization, and final summarization. All the above procedures were performed via the RMA algorithm in GeneSpring Gx12.1 to evaluate differential gene expression and clustering analysis. The ratio of the geometric means of the expression analysis of the relevant gene fragments was computed to yield a fold change analysis. Confidence intervals and corrected p-values were calculated using a Rank Wilcoxson test. Statistical significance was assessed by considering the false discovery rate (FDR), which accounts for multiple testing. Only genes with at least a two-fold difference between the ART-IF and ART-R with a *p*-value of <0.05 were considered as significantly differentially expressed. Heat maps showing differential expression were generated. For pathway analysis of the significantly expressed genes, the Database for Annotation, Visualization and Integrated Discovery (DAVID) (https://david.ncifcrf.gov/) and Panther (http://www.pantherdb.org/) software were used. Protein-protein interactions of the shortlisted genes were analyzed using STRING 9.1 software (http://string-db.org/). Microarray raw and processed data was deposited in the Gene Expression Omnibus (GEO) database [19] in the National Center for Biotechnology Information (NCBI) and are accessible through GEO Series accession number GSE77939 (https://www.ncbi.nlm.nih.gov/geo/query/acc.cgi?acc=GSE77939).

### 2.7 Quantitative real-time PCR (qRT-PCR)

The differentially expressed genes shortlisted from microarray analysis were validated on a replication cohort of 10 samples in each group (in triplicate) by quantitative real-time PCR (qRT-PCR) using Light Cycler 480 (Roche, Branchburg, NJ, USA). Briefly, RNA was extracted using the Qiagen PaxGene RNA isolation kit, converted into cDNA using a ProtoScript^®^ cDNA Synthesis kit (NEB, USA) and quantitative real-time PCR was performed with gene-specific primers as indicated in S1 Table. Relative quantitation (fold change) was calculated by ΔCt values obtained after normalization with the expression of GAPDH.

### 2.8 CD4 count and plasma viral load

The CD4^+^ T cells were enumerated by a bead-based flow-cytometry kit (Trucount^™^, BD Biosciences Immunocytometry Systems, San Jose, CA, USA) and the plasma viral load was estimated with the Cobas Taqman HIV-1 test, version 2.0 (Roche, Branchburg, NJ, USA) according to manufacturer’s protocol.

### 2.9 Statistical analyses

Statistical analysis was performed using Graph Pad Prism (version 5.0) and SPSS (version 19.0). Discrete and continuous variables were compared between cases and control subjects using the unpaired t-test, one-way ANOVA, and Mann-Whitney test as appropriate. A two-sided *p*-value of <0.05 was considered statistically significant. Validation of qRT-PCR data is presented in terms of fold change in the expression of shortlisted genes in a comparison between the different study groups and healthy controls, after normalizing ΔC_T_ values with the housekeeping gene (GAPDH), fold change was determined via the 2^-ΔΔCT^ values [20].

## 3 Results

We screened whole-genome expression profiles from a total of 30 subjects in the discovery cohort, this was comprised of: 25 subjects who were HIV-1 subtype-C seropositive and 5 healthy volunteers as control. Among HIV-positive individuals, 5 were ART-naïve and 20 subjects were on ART for at least one year. Among ART-experienced individuals, 10 individuals had responded to therapy showing undetectable viral loads and their CD4^+^ T cell count recovered to >450 cells/μL and were termed as ART-responders (ART-R), while 10 individuals having undetectable viral loads (<400 copies/mL) but their CD4^+^ T counts remaining below 250 cells/μL, were categorized as ‘immunological failures’ (ART-IF). The microarray data from study subjects after deep analysis is presented hereafter and comparisons of gene expression profiles among different study groups determined to find the key differences.

### 3.1 Demographic details of the study population

The clinical profile for each study group from the discovery cohort is summarized in Table 1. For HIV-1 seropositive individuals, we obtained the baseline values for CD4^+^ T cell count, at the time of diagnosis of HIV-1, the start of ART, and at the time of recruitment in this study. At the start of therapy, the ART-R group had a slightly higher median CD4^+^ T cell count as compared to ART-IF, but the difference was not statistically significant. Plasma viral load was <400 copies/mL at the time of recruitment in all the patients undergoing therapy (on ART), but mean CD4 count remained <250 in ART-IF group (202 cells/μL), while it improved to 495 cells/μL in ART-R group. The majority of individual’s route of infection was due to unprotected sex.

**Table 1:** Demographic & clinical details of the discovery cohort analyzed for gene expression profiling by microarray.

Study Characteristic	HCs^#^ (n = 5)	ART-IF* (n = 10)	ART-R^├^ (n = 10)	ART-N^┼^ (n = 5)
Age (years) (median, range)	37 (32–54)	36 (32–40)	35 (30–42)	37 (34–39)
Duration of HIV-1 infection in months) (mean, range)	-	24 (16–34)	26 (18–46)	22 (14–38)
CD4^+^ T cell count (cells/μL) (median and range) when diagnosed for HIV	-	717 (517–933)	783 (419–1116)	688 (499–987)
CD4^+^ T cell count (cells/μL) (median and range) when therapy started	-	180 (100–240)	192 (95–260)	-
CD4^+^ T cell count (cells/μL) (median and range) at the time of recruitment	862.5 (463–1471)	202 (150–247)	495 (411–884)	410 (225–804)
Average time for initiation of HAART (In years) (median and range)	-	1.6 (1.2–2.4)	1.8 (1.3–2.2)	-
Plasma HIV-1 RNA (copies/mL)	-	<450	<450	
**Ongoing HAART regimen in patients (%)**				-
NRTI (3TC and AZT) + NNRT (NVP)	-	4 (57.15)	3 (0.60)	-
NRTI (3TC and d4T) + NNRT (NVP)	-	3 (42.85)	2 (0.40)	-
**Exposure to HIV-1 History**				
Sexual	-	88 (%)	83 (%)	86 (%)
Blood Transfusion	-	2 (%)	4 (%)	4 (%)
Intravenous Drug User	-	3 (%)	3 (%)	4 (%)
Other	-	7 (%)	10 (%)	6 (%)

NRTI (Nucleoside reverse transcriptase inhibitors) 3TC (Lamivudine), AZT (Zidovudine), d4T (Stavudine)

NNRTI (Non-nucleoside reverse transcriptase inhibitors) NVP (Nevirapine)

### 3.2 Gene expression analysis results of microarray

**Fig 1:**
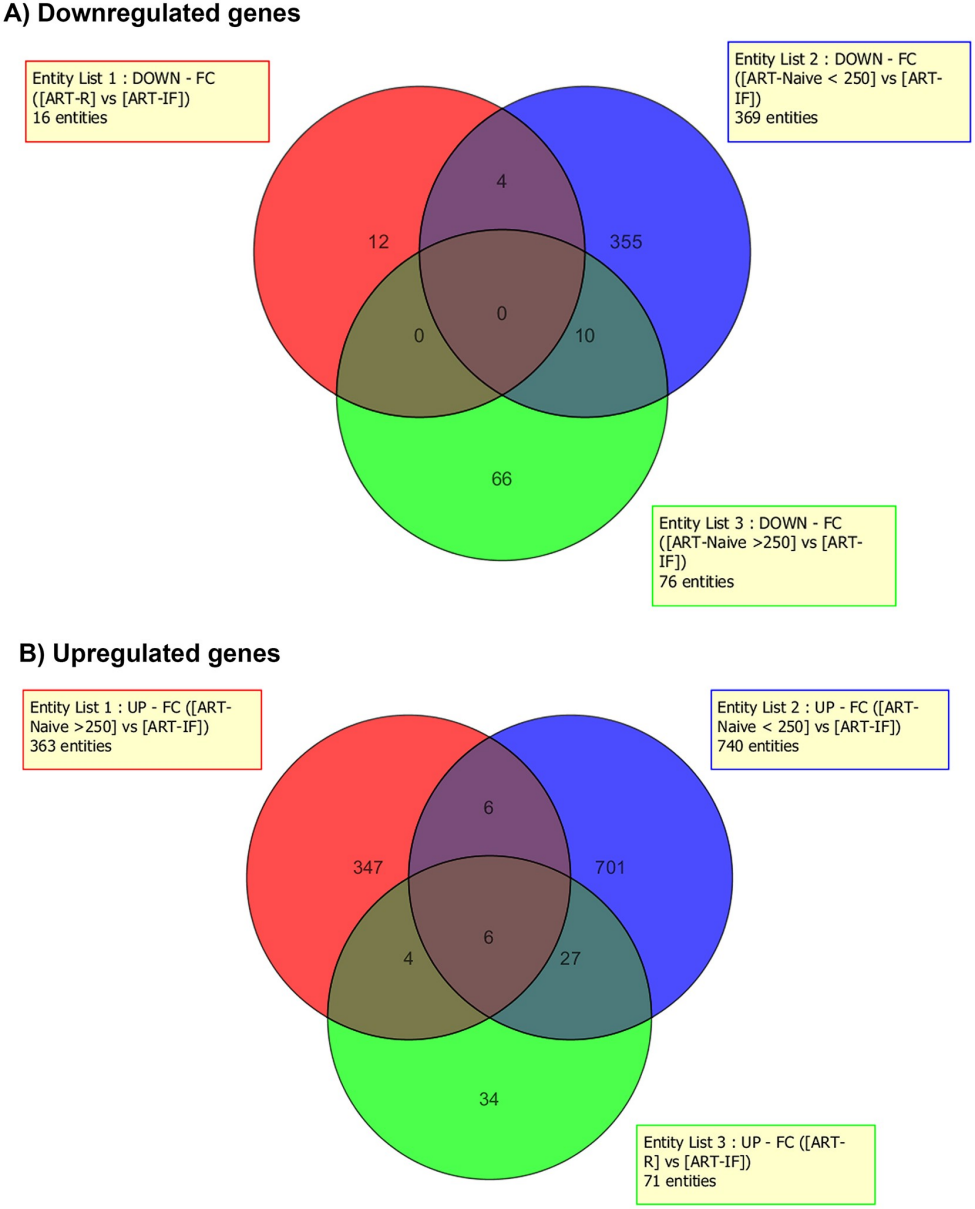
Venn diagram showing the downregulated and upregulated genes. Briefly, among HIV infected, overall, 16 probe sets (10 genes) significantly up-regulated and 71 probe sets (60 genes) down-regulated (>2-fold change) in ART-IF as compared to ART-R.

**Fig 2:**
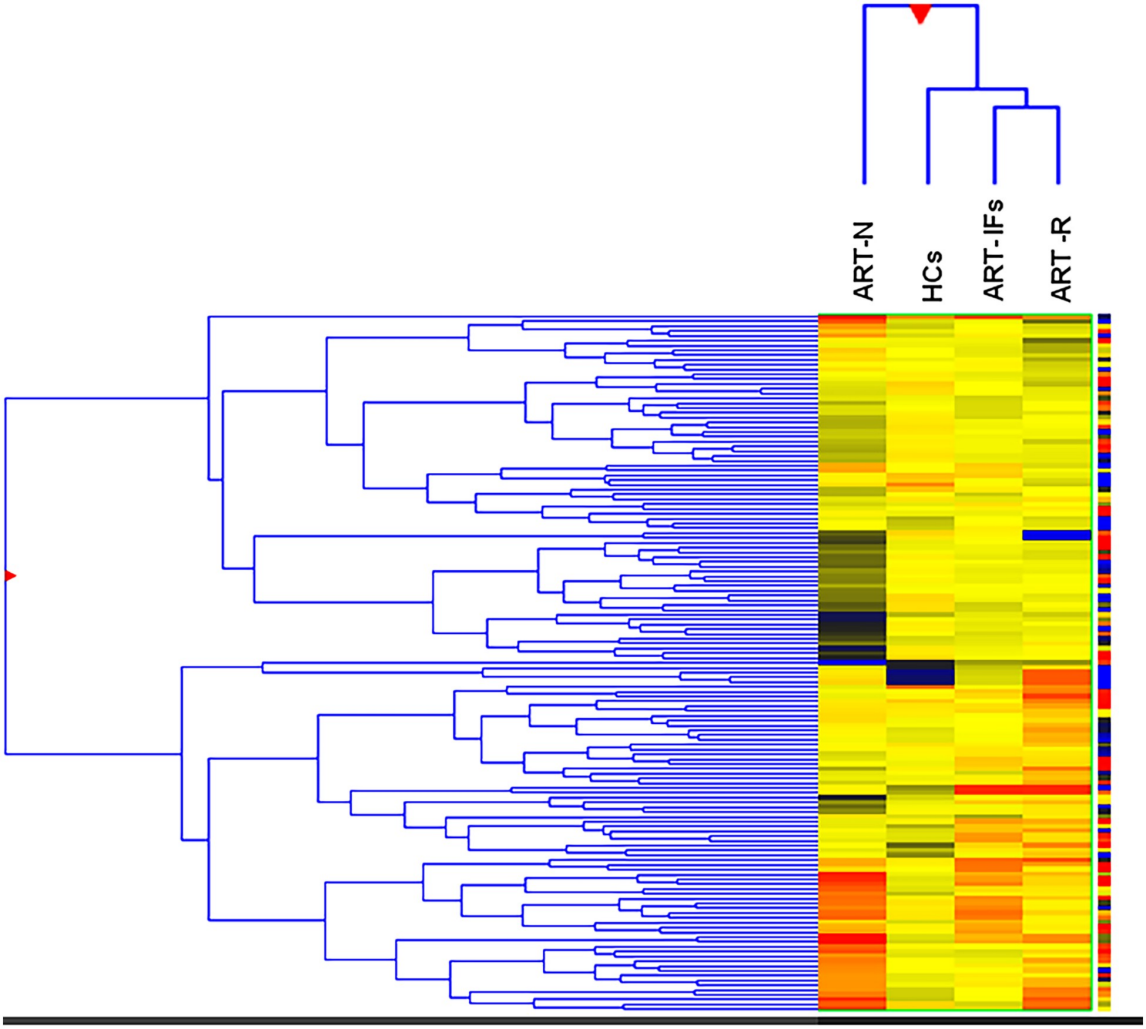
Heatmap and hierarchical clustering of genes in studied groups. Hierarchical clustering (Hcl) expression image is shown. It is also clear from the above Hcl expression image i.e. ART-IF and ART-R clustered together. Red color shows over-expressed genes (>2.0) & Blue color shows under-expressed genes (<2.0). (The Hcl expression plot has been generated based on log2 normalized intensity value).

**Fig 3:**
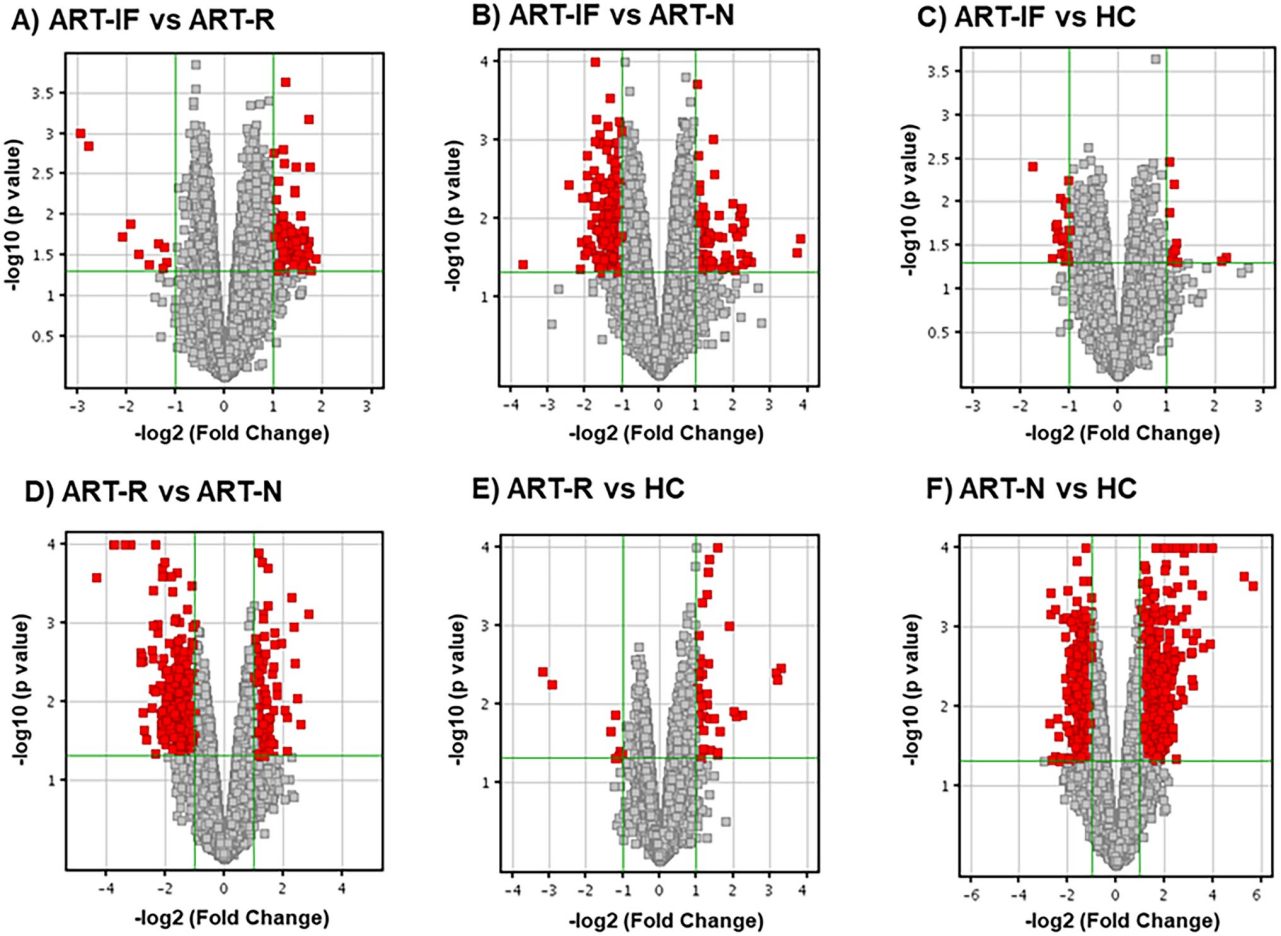
Volcano plots showing the distribution of significantly upregulated and downregulated genes. Volcano plots were constructed by plotting the negative log of the p-value on the y-axis (-log10). The x-axis is the log of the log fold change among different groups. Distribution of downregulated and upregulated genes among ART-IF, ART-R, ART-N, and HCs.

The gene expression profiling by microarray revealed up-regulation of 553 probe sets and down-regulation of 568 probe sets (>2-fold change) in HIV-1 infected group as compared to HCs. Among HIV infected, we found overall 16 probe sets (10 genes) were significantly up-regulated and 71 probe sets (60 genes) down-regulated (>2-fold change) in ART-IF as compared to ART-R (Tables 2 and 3). The common genes expressed among different study groups are shown in Venn diagrams (Fig 1). The hierarchical clustering image (Fig 2) revealed a differential clustering of HIV-seropositive therapy-naive individuals, HIV therapy groups, and HCs. It is also clear from the hierarchical clustering expression image that ART-IF and ART-R are clustered together. The distribution of downregulated and upregulated genes among ART-IF, ART-R, and ART-N groups is shown in the volcano plots (Fig 3).

**Table 2:** Summary of genes differentially up-regulated in ART-IF as compared to ART-R.

Sr. No.	Probe Set ID	P value	FC (abs)	Gene Symbol
1	200032_PM_s_at	0.019476	2.296302	*RPL9*
2	200099_PM_s_at	0.034967	2.447483	*RPS3A /// SNORD73A*
3	11715237_s_at	0.019046	2.734538	*RPL9 /// TIPIN*
4	11715238_x_at	0.027669	2.662893	*RPL9*
5	11715385_s_at	0.0265	2.675089	*C4orf46 /// TOMM7*
**6**	**11715694_at**	**0.01502**	**3.114485**	***COX7B***
7	11715695_x_at	0.020819	2.720116	*COX7B*
8	11716291_s_at	0.040746	2.052408	*HNRNPA0*
9	11716320_x_at	0.035174	2.385255	*RPS3A /// SNORD73A*
10	11716730_a_at	0.016864	2.141748	*ERH*
11	11717235_s_at	0.044171	2.760956	*RPS7*
12	11718051_a_at	0.004063	2.419594	*RPL27*
13	11718735_at	0.032536	2.026552	*PCMTD1*
14	11718886_a_at	0.018573	2.115311	*TPRKB*
15	11720847_s_at	0.03754	2.034386	*PHF6*
16	11720952_a_at	0.003438	2.084141	*DCUN1D4*
17	11722393_s_at	0.014836	2.007706	*TMEM126B*
18	11722804_x_at	0.043867	2.071918	*PTBP2*
19	11723280_at	0.002404	2.144142	*CREBZF*
20	11723429_s_at	0.037912	2.660196	*TPP2*
21	11723957_x_at	0.012323	2.549908	*LSM5*
22	11724120_a_at	0.005264	2.099239	*TRIM59*
23	11724886_a_at	0.002174	2.882279	*LRRN3*
24	11726333_s_at	0.005183	2.420848	*LEF1*
25	11726515_a_at	0.021745	2.023199	*CLK4*
**26**	**11727510_at**	**0.04954**	**3.010162**	***UBN2***
27	11727834_at	0.021761	2.256306	*WDR43*
28	11727860_at	0.017688	2.23923	*TMEM209*
29	11728645_a_at	0.023682	2.598158	*NUCKS1*
30	11728646_at	0.021583	2.507985	*NUCKS1*
31	11729696_at	0.004161	2.227162	*C2orf69*
32	11730444_s_at	0.027764	2.289883	*CCNJ*
33	11730890_at	0.034229	2.175925	*NSUN6*
34	11730906_at	0.039818	2.016503	*PDS5B*
35	11731493_at	0.01396	2.10411	*ITCH*
36	11733216_s_at	5.89E-05	2.495168	*USP53*
37	11733353_at	0.010017	2.270333	*CRTAM*
38	11734051_a_at	0.009705	2.342362	*TRAT1*
39	11734882_a_at	0.042899	2.321562	*ARID2*
**40**	**11735394_s_at**	**9.76E-04**	**2.941312**	***XCL1 /// XCL2***
41	11736498_a_at	0.045336	2.44936	*TNRC6B*
42	11737237_a_at	0.006182	2.103122	*EOMES*
43	11739568_a_at	0.014809	2.341502	*ZCCHC7*
44	11739860_a_at	0.020893	2.413266	*ITGA6*
45	11740670_a_at	0.038805	2.458961	*TNRC6B*
46	11741013_a_at	0.002266	2.939714	*LRRN3*
47	11741059_s_at	0.036282	2.114344	*SATB1*
48	11741640_a_at	0.035141	2.088273	*ZFX*
49	11741813_s_at	0.019605	2.324938	*HINT1*
50	11743530_a_at	0.021704	2.131954	*CENPK*
51	11744411_s_at	0.010599	2.6119	*RSL24D1*
52	11744730_s_at	0.006604	2.190669	*DLEU2 /// DLEU2L*
53	11745624_s_at	0.006049	2.159883	*INADL*
54	11749630_a_at	0.009842	2.089378	*KRR1*
55	11750091_a_at	0.012544	2.181014	*TPRKB*
56	11750694_a_at	0.045489	2.098371	*RECQL*
57	11751867_a_at	0.041369	2.381404	*C8orf59*
58	11753350_x_at	0.019908	2.297098	*KRR1*
59	11753656_x_at	0.015743	2.074655	*RPS15A*
60	11753694_x_at	0.030696	2.084997	*RPS15A*
61	11754933_s_at	0.038586	2.156223	*TSC22D2*
62	11757027_x_at	0.047178	2.709783	*RPL31*
63	11757328_x_at	0.005298	2.036332	*RPL27*
64	11757422_x_at	0.011039	3.374636	*RPL23*
65	11757507_s_at	0.012835	2.021164	*DSP*
66	11757673_x_at	0.046793	2.113096	*RPL39*
67	11757785_x_at	0.048919	2.004613	*HMGB1*
68	11758357_x_at	0.043301	2.281361	*RPL9*
69	11758633_s_at	0.005768	2.406391	*PDCD5*
70	11759196_at	0.032013	2.046226	*METTL10*
71	11760182_at	0.001744	2.264548	*ZNF549*

**Table 3:** Summary of genes differentially down-regulated in ART-IF as compared to ART-R.

Sr. No.	Probe Set ID	P value	FC (abs)	Gene Symbol
1	200032_PM_s_at	0.019476	2.296302	*RPL9*
2	200099_PM_s_at	0.034967	2.447483	*RPS3A /// SNORD73A*
3	11715237_s_at	0.019046	2.734538	*RPL9 /// TIPIN*
4	11715238_x_at	0.027669	2.662893	*RPL9*
5	11715385_s_at	0.0265	2.675089	*C4orf46 /// TOMM7*
**6**	**11715694_at**	**0.01502**	**3.114485**	***COX7B***
7	11715695_x_at	0.020819	2.720116	*COX7B*
8	11716291_s_at	0.040746	2.052408	*HNRNPA0*
9	11716320_x_at	0.035174	2.385255	*RPS3A /// SNORD73A*
10	11716730_a_at	0.016864	2.141748	*ERH*
11	11717235_s_at	0.044171	2.760956	*RPS7*
12	11718051_a_at	0.004063	2.419594	*RPL27*
13	11718735_at	0.032536	2.026552	*PCMTD1*
14	11718886_a_at	0.018573	2.115311	*TPRKB*
15	11720847_s_at	0.03754	2.034386	*PHF6*
16	11720952_a_at	0.003438	2.084141	*DCUN1D4*
17	11722393_s_at	0.014836	2.007706	*TMEM126B*
18	11722804_x_at	0.043867	2.071918	*PTBP2*
19	11723280_at	0.002404	2.144142	*CREBZF*
20	11723429_s_at	0.037912	2.660196	*TPP2*
21	11723957_x_at	0.012323	2.549908	*LSM5*
22	11724120_a_at	0.005264	2.099239	*TRIM59*
23	11724886_a_at	0.002174	2.882279	*LRRN3*
24	11726333_s_at	0.005183	2.420848	*LEF1*
25	11726515_a_at	0.021745	2.023199	*CLK4*
**26**	**11727510_at**	**0.04954**	**3.010162**	***UBN2***
27	11727834_at	0.021761	2.256306	*WDR43*
28	11727860_at	0.017688	2.23923	*TMEM209*
29	11728645_a_at	0.023682	2.598158	*NUCKS1*
30	11728646_at	0.021583	2.507985	*NUCKS1*
31	11729696_at	0.004161	2.227162	*C2orf69*
32	11730444_s_at	0.027764	2.289883	*CCNJ*
33	11730890_at	0.034229	2.175925	*NSUN6*
34	11730906_at	0.039818	2.016503	*PDS5B*
35	11731493_at	0.01396	2.10411	*ITCH*
36	11733216_s_at	5.89E-05	2.495168	*USP53*
37	11733353_at	0.010017	2.270333	*CRTAM*
38	11734051_a_at	0.009705	2.342362	*TRAT1*
39	11734882_a_at	0.042899	2.321562	*ARID2*
**40**	**11735394_s_at**	**9.76E-04**	**2.941312**	***XCL1 /// XCL2***
41	11736498_a_at	0.045336	2.44936	*TNRC6B*
42	11737237_a_at	0.006182	2.103122	*EOMES*
43	11739568_a_at	0.014809	2.341502	*ZCCHC7*
44	11739860_a_at	0.020893	2.413266	*ITGA6*
45	11740670_a_at	0.038805	2.458961	*TNRC6B*
46	11741013_a_at	0.002266	2.939714	*LRRN3*
47	11741059_s_at	0.036282	2.114344	*SATB1*
48	11741640_a_at	0.035141	2.088273	*ZFX*
49	11741813_s_at	0.019605	2.324938	*HINT1*
50	11743530_a_at	0.021704	2.131954	*CENPK*
51	11744411_s_at	0.010599	2.6119	*RSL24D1*
52	11744730_s_at	0.006604	2.190669	*DLEU2 /// DLEU2L*
53	11745624_s_at	0.006049	2.159883	*INADL*
54	11749630_a_at	0.009842	2.089378	*KRR1*
55	11750091_a_at	0.012544	2.181014	*TPRKB*
56	11750694_a_at	0.045489	2.098371	*RECQL*
57	11751867_a_at	0.041369	2.381404	*C8orf59*
58	11753350_x_at	0.019908	2.297098	*KRR1*
59	11753656_x_at	0.015743	2.074655	*RPS15A*
60	11753694_x_at	0.030696	2.084997	*RPS15A*
61	11754933_s_at	0.038586	2.156223	*TSC22D2*
62	11757027_x_at	0.047178	2.709783	*RPL31*
63	11757328_x_at	0.005298	2.036332	*RPL27*
64	11757422_x_at	0.011039	3.374636	*RPL23*
65	11757507_s_at	0.012835	2.021164	*DSP*
66	11757673_x_at	0.046793	2.113096	*RPL39*
67	11757785_x_at	0.048919	2.004613	*HMGB1*
68	11758357_x_at	0.043301	2.281361	*RPL9*
69	11758633_s_at	0.005768	2.406391	*PDCD5*
70	11759196_at	0.032013	2.046226	*METTL10*
71	11760182_at	0.001744	2.264548	*ZNF549*

### 3.3 Pathway analysis of shortlisted genes

**Fig 4:**
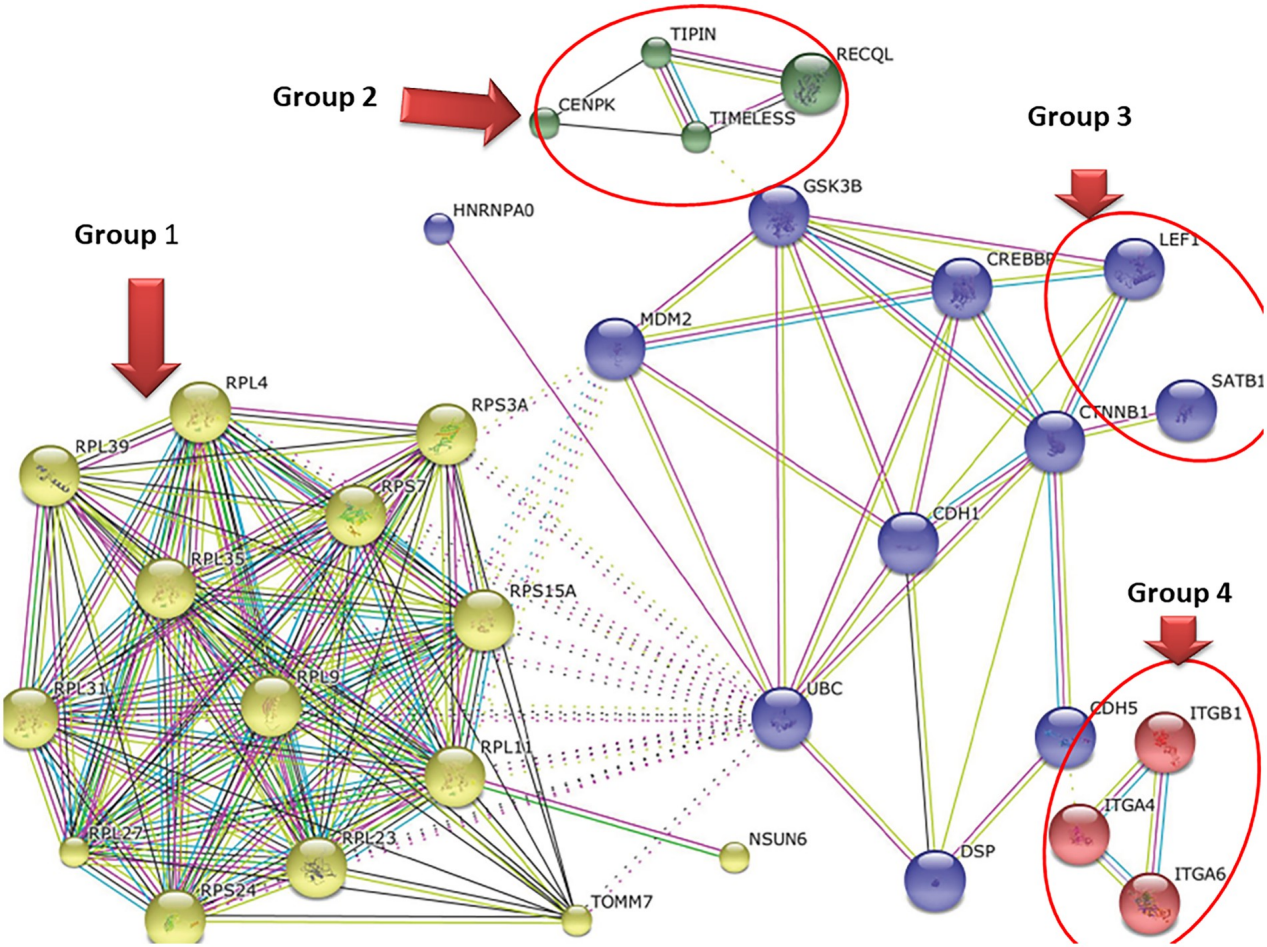
Interactive network of the regulatory molecules. String analysis was performed to study the protein-protein interaction. Downregulation of proteins differentially expressed in immunological failure HIV-1 subtype-C patients as compared to ART-R. Briefly, protein-protein interaction revealed four clusters with distinct biological functions. **Group 1**: Molecules involved in viral transcription, viral genome expression, and translation termination or inhibition. **Group 2**: Molecule involved in the cell cycle process. **Group3**: CD4-positive, alpha-beta T-cell differentiation, and activation. *Lymphoid enhancer-binding factor 1 (LEF-1)*. **Group 4**: Integrin-mediated signaling pathway.

Protein-protein interactions and subsequent pathways affected by the upregulated genes in ART-IF as compared to ART-R group were analyzed using the online software STRING 9.1, which revealed four major clusters of genes (Group-1, Group-2, Group 3 and Group 4) involved primarily in immunological-non responsiveness (Fig 4). Group-1 genes are primarily involved in viral transcription, and translation termination. This pathway explains the low viral load in ART-IF subjects, but it also implies an overall lowering of the cellular biological processes (transcription & translation) within this group. Genes up-regulated in the ART-IF group were primarily those involved in the cell cycle phase specifically gene that regulates the nuclear division and the mitotic cell cycle (Group 2 genes), CD4+α/βT-cell differentiation, and activation. *Lymphoid enhancer-binding factor 1 (LEF-1)* is a downstream effector of the Wnt signal transduction pathway and is involved in the regulation of T cell development in the thymus [21]. These are also expressed in mature peripheral primary T cells, but their expression is down-regulated following T cell activation. Although the decisive roles of LEF-1 and TCF-1 (T cell factor-1) in the early stages of T cell development are well documented, the functions of these factors in mature peripheral T cells are not yet well understood (Group 3 genes). Integrin-mediated signaling pathway, subunit α6 (CD49f), a cell-surface molecule plays an important role in both mesenchymal stem cell (MSC) sphere formation and differentiation potential. CD49f promoter acts as a scaffold for the pluripotency factors OCT4 and SOX2 and also activates the PI3K/AKT/p53 pathway [22]. Therefore, since it contributes to the maintenance of pluripotency in human embryonic stem cells (hESCs), it indicates the dysfunctional stem cells in ART-IF, resulting in a reduced turnover of CD4^+^ T cells, and warrants further investigations (Group 4).

Furthermore, we used online software ‘Panther’ to generate a list of diseases affected by these differentially up-regulated genes in ART-IF individuals, based on their molecular function or participation in identical biological processes or pathways. Analyzed data indicated that these genes participate in cytoskeletal regulation by Rho GTPase and Huntington disease. Similarly, the differentially up-regulated genes analyzed in ART-IF revealed genes involved in enkephalin release, heterotrimeric G-protein signaling pathway-Gi alpha and Gs alpha-mediated pathway, cadherin signaling pathway, integrin signaling pathway, Wnt signaling pathway, gonadotropin-releasing hormone receptor pathway, general transcription by RNA polymerase I, Purine metabolism, T-cell activation, p38 MAPK pathway, pyrimidine metabolism, p53 pathway, transcription regulation by bZIP transcription factor and apoptosis signaling pathway, which may have some association with immunological failure in HIV-infected and treated individuals.

### 3.4 Validation of microarray data

Twenty genes were shortlisted based on differentially expressed genes from microarray analysis for validation by quantitative real-time PCR (qRT-PCR) (S2 Table). Validation was performed on a replication cohort of 40 samples (10 subjects in each age and sex-matched group in triplicate). The reference sequences for the genes were downloaded from the NCBI and were used for designing the primers for the expression analysis (S1 Table).

**Fig 5:**
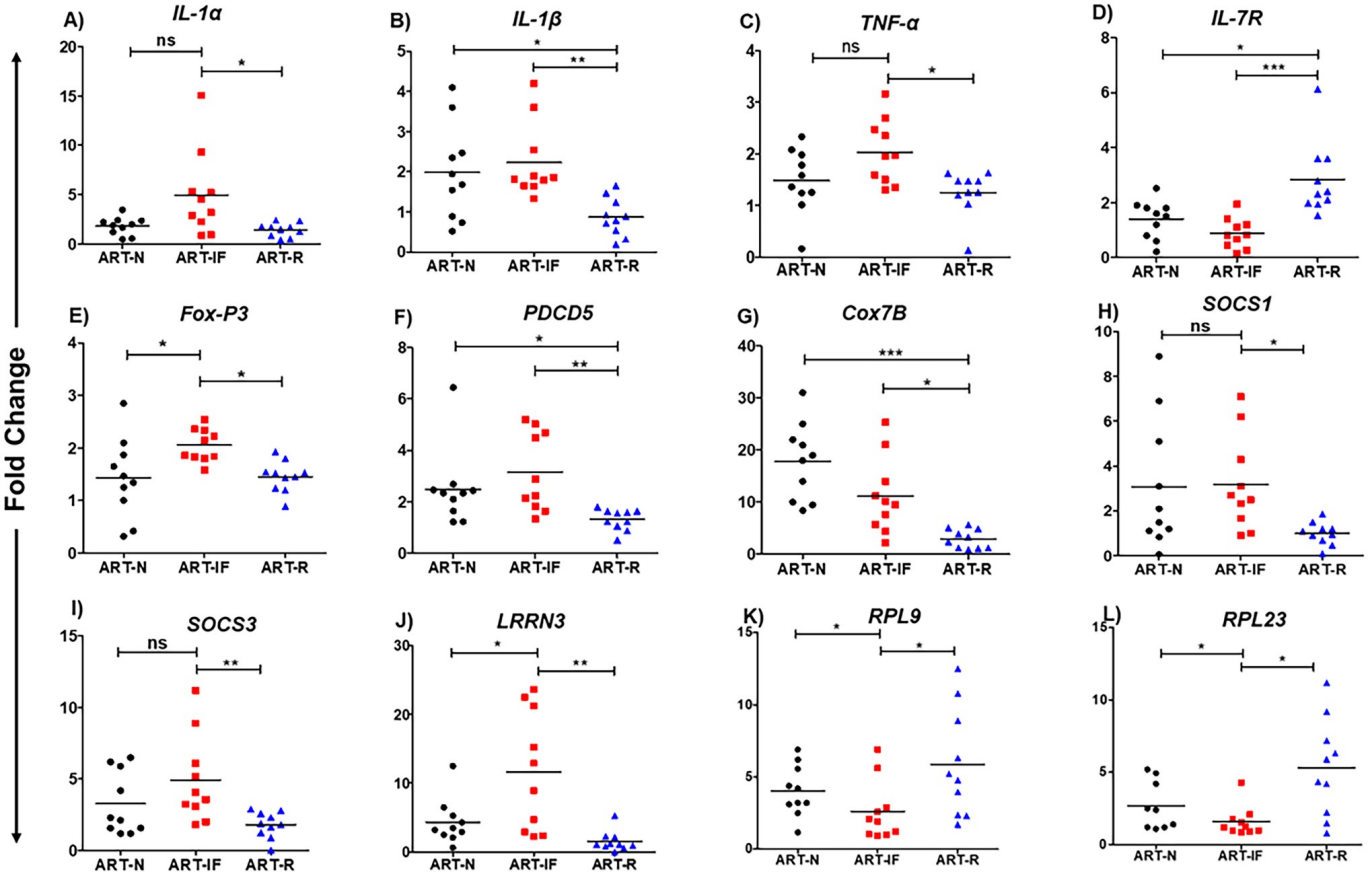
qRT-PCR analysis and validation of microarray gene expression data. Differentially expressed genes among study groups (ART-IF, ART-R, and ART-N) were shortlisted for validation. Scatter plots showing the fold change in expression profile for *IL-1α*, *IL-1β*, *IL-7R*, *TNF-α*, *FoxP3*, *PDCD5*, *COX7B*, *SOCS1*, *SOCS3*, *LRRN3*, *RPL9*, and *RPL23* genes by qRT-PCR (n = 10 each group). Significant values are indicated by star (*p<0.05, **p<0.01, ***p<0.001).

We compared relative mRNA expression from the twenty selected genes by qRT-PCR and found statistically significant differences among the various study groups for twelve genes *IL-1α*, *IL-1β*, *IL-7R*, *TNF-α*, *FoxP3*, *PDCD5*, *COX7B*, *SOCS1*, *SOCS3*, *RPL9*, *RPL23*, and *LRRN3* respectively (Fig 5). Expression profiles of the other eight genes (*TUBB2A*, *RHD*, *RPL27*, *RSL24D1*, *IL-7*, *LSM5*, *KRR1* and *PDCD1*, respectively) selected for validation in the replication cohort were not significantly different among these groups. Among the pro-inflammatory cytokine genes, *IL-1α*, *IL-1β*, and *TNF-α* were found to have significantly increased expression in ART-IF as compared to ART-R, and ART-N (Fig 5A, 5B and 5C). The validated data indicated a significantly lower expression *IL-7R* in ART-IF as compared to the ART-R group (Fig 5D). *FoxP3* expression increased significantly in ART-IF as compared to ART-R as well as ART-N (Fig 5E). The expression of *PDCD5*, which is known to promote programmed cell death, was significantly higher in ART-IF as compared to ART-R (Fig 5F). More notably, the expression of immune regulatory genes *Cox7B*, *SOCS1*, *SOCS3*, and *LRRN1* were also significantly upregulated in ART-IF compared to ART-R, indicating profound immune activation despite upregulation of negative regulators associated with immunological non-responsiveness (Fig 5G, 5H, 5I and 5J). Genes associated with ribosomal biogenesis were significantly lower among ART-IF compared to the ART-R group (Fig 5K and 5L). Altogether, qRT-PCR validation data demonstrates significant differences in key genes associated with T cell homeostasis, immune activation, apoptosis, and immune-regulation pathways.

## 4 Discussion

The availability and success of antiretroviral therapy (ART) has increased the life expectancy of HIV patients, but one major challenge that remains is the inability to restore normal immunologic health in patients having a discordant response. Suboptimal CD4^+^ T cell pool reconstitution in response to ART has been associated with a substantial increase in the risk of AIDS-related mortality and morbidity. In the present study, we evaluated the possible link between immunological non-responsiveness to HAART and whole-genome expression profiling of unique HIV-1 infected cohort showing signs and symptoms of immunological failure vis-a-vis those who responded to the treatment in terms of CD4^+^ T-cell gain after one year of ART.

We found significantly different gene expression profile among different study groups. Hierarchical clustering showed major differences in the gene expression among HIV-infected naive compared to HCs. Advanced analysis of the data revealed 10 genes were significantly up-regulated and 60 genes down-regulated in ART-IF as compared to ART-R (Fig 1). Although, ART-IF and ART-R gene expression profiles were different, yet the two groups clustered together in hierarchical clustering (Fig 3). This might be due to some common features like complete viral suppression post-ART among these two groups. Further, based on the differential expression linked with functions and pathway analysis for protein-protein interaction, some genes were shortlisted for validation. Among these, we found statistically significant differences in expression levels of *IL-1α*, *IL-1β*, *IL-7R*, *TNF-α*, *FoxP3*, *PDCD5*, *COX7B*, *SOCS1*, *SOCS3*, *RPL9*, *RPL23*, and *LRRN3* genes among ART-IF compared to ART-R, confirming their intimate relationship with immunological response to therapy. Nevertheless, host genetic factor studies are still needed to determine the role of variations in immunological non-responsiveness.

Altogether, our microarray data and qRT-PCR validation results indicated that the expression of some key genes involved in the regulation of T cell homeostasis, immune activation, pro-inflammatory cytokine production, apoptosis, and immune-regulatory are possibly associated with the major mechanisms of immunological non-responsiveness in ART-IF groups. It is now clear that the HIV persists in gut-associated and other lymph nodes despite long-term ART, and this persistent viral replication in lymphoid tissues may be the proximal cause of chronic immune activation during the progression of the disease. This suggests that the ART-IF may have a larger latent reservoir pool of HIV compared to those who respond to therapy. A Previous study has shown that lymphoid fibrosis in SIV may cause an insufficient or ineffective CD4^+^ T cell recovery and anti-fibrotic therapy improved immune reconstitution post-therapy [23]. Thus, the combined effect of increased immune activation, altered T cell homeostasis, increased microbial translocation, and continuous damage and fibrosis in the gut mucosa of immunological non-responders may be preventing the full CD4^+^ T cell recovery in these patients. Immunological activity is controlled by a balance between proliferation and apoptotic cell death of CD4^+^ T cells. Our data shows enhanced cell death pathway genes among ART-IF. Besides, the genes linked to proliferation such as Integrin-mediated signaling pathway genes shows dysregulation in ART-IF. This pathway is known to play a crucial role in the maintenance of pluripotency in human embryonic stem cells, indicating the dysfunctional stem cells in ART-IF, resulting in overall reduced turnover of CD4^+^ T cells. Therefore, in future mechanisms investigating impaired maintenances and cycling of CD4 T lymphocytes are warranted.

In current literature, there is only a limited number of studies on the mechanisms of immunological non-responsiveness. Previous studies have shown that the FoxP3 and single nucleotide polymorphisms (SNPs) in the *IL-7R* gene play a critical role in CD4^+^ T cell recovery [24, 25]. The expression of TIM-3 in NK cells has also been shown to affect the immunological responsiveness to therapy [26]. Recently, two studies have shown the critical role of gut damage and the size of the HIV reservoir in immunological non-responsiveness to anti-HIV therapy [27, 28]. More recently, upregulation of NLRP3 and caspase-1 is observed in ART-IF patients, indicating the role of persistent immune activation and pyroptosis could induce CD4 T-cell loss [29]. All these studies suggest that various host-mediated factors could affect the discordant immunological response to therapy. The results of our comprehensive microarray-based study corroborated and therefore validated previously reported findings, elucidating the mechanisms of non-responsiveness to therapy in a small but definitive population among HIV-1 subtype-C infected individuals. The study clearly indicates that deranged homeostasis due to low expression of IL-7 receptors coupled with increased apoptosis contributes to immunological non-responsiveness in a small population of patients who are put on ART. The differences could be because of epigenetic changes, which may be dynamic though, but cannot be ruled out in these patients post one year of anti-retroviral treatment. The findings may lead to the design of novel molecules that may help in better management of disease in such patients. However, further functional studies are needed to better understand the precise molecular mechanisms that may help define strategies to modulate pathways involved in immunologic non-responsiveness. This will have huge translational implications and could potentially help in designing “specific therapies” better suited for such patients. The major strength of the study is the recruitment of a unique and previously not well-studied cohort of predominantly HIV-1 subtype-C-infected individuals from North Indian territory. In our cohort, the CD4^+^ T counts at the start of therapy were the same, but one limitation was a lack of viral loads monitoring at the start of therapy. For further validation of this data in the future, multi-centric studies focused on the functional validation of immunological non-responsive genes in well-characterized cohorts are warranted.

## Supporting information

S1 Table Microarray data validation primers. The following primers were used in the qPCR validation experiments.
(DOCX)

S2 Table Summary of shortlisted genes. The following genes were shortlisted after an in-depth analysis for the validation of microarray data.
(DOCX)
