# Supplementary material for: Signature genes associated with immunological non-responsiveness to anti-retroviral therapy in HIV-1 subtype-c infection
Source: PLoS One. 2020 Jun 24;15(6):e0234270. doi: 10.1371/journal.pone.0234270 (PMC7313746; doi:10.1371/journal.pone.0234270)
Supplement: S1 Table — The following primers were used in the qPCR validation experiments. (DOCX) [file pone.0234270.s001.docx]

**Table S1:** Summary of shortlisted genes used for validation of microarray data

| **Sr. No.** | **Gene Symbol** | **Name of the Gene** |
| --- | --- | --- |
| 1 | *TUBB2A* | Tubulin, Beta 2A Class IIa |
| 2 | *RHD* | Rh Blood Group, D Antigen |
| 3 | *RPL9* | Ribosomal Protein L9 |
| 4 | *COX7B* | Cytochrome C Oxidase Subunit VIIb |
| 5 | *RPL27* | Ribosomal Protein L27 |
| 6 | *RPL23* | Ribosomal Protein L23 |
| 7 | *RSL24D1* | Ribosomal L24 Domain Containing 1 |
| 8 | *LSM5* | LSM5 Homolog, U6 Small Nuclear RNA Associated (S. Cerevisiae) |
| 9 | *LRRN3* | Leucine Rich Repeat Neuronal |
| 10 | *PDCD5* | Programmed Cell Death 5 |
| 11 | *KRR1* | KRR1, Small Subunit (SSU) Processome Component, Homolog (Yeast) |
| 12 | *IL7* | Interleukin 7 |
| 13 | *IL7R* | Interleukin 7 receptor |
| 14 | *IL1A* | Interleukin 1A |
| 15 | *IL1B* | Interleukin 1B |
| 16 | *PDCD1* | Programmed Cell Death 1 |
| 17 | *TNF* | Tumor necrosis factors |
| 18 | *SOCS1* | Suppressor of cytokine signaling 1 |
| 19 | *SOCS3* | Suppressor of cytokine signaling 3 |
| 20 | *FOXP3* | Forkhead box P3 |
