# Supplementary material for: Signature genes associated with immunological non-responsiveness to anti-retroviral therapy in HIV-1 subtype-c infection
Source: PLoS One. 2020 Jun 24;15(6):e0234270. doi: 10.1371/journal.pone.0234270 (PMC7313746; doi:10.1371/journal.pone.0234270)
Supplement: S2 Table — The following genes were shortlisted after an in-depth analysis for the validation of microarray data. (DOCX) [file pone.0234270.s002.docx]

**Table S2:** The following primers are finalized to be used in the PCR-array experiments.

| **Serial No.** | **Primer ID** | **Primer Sequence 5' to 3'** |
| --- | --- | --- |
| 1 | TUBB2A For | AAT ATG TAC CTC GGG CCA TC |
|  | TUBB2A Rev | TCT GAC TCC TTC CTC ACC AC |
| 2 | RHD For | GAC AGC TTT AGG CAA CCT GA |
|  | RHD Rev | GCA GAC AAA CTG GGT ATC GT |
| 3 | RPL9 For | CTG GCT ACC GTT CGG ACT AT |
|  | RPL9 Rev | GCA ACA CCT GGT CTC ATC C |
| 4 | COX7B For | ATC GTC TCC AAG TTC GAA GC |
|  | COX7B Rev | TTT GGG GTA ACT CTG CCA AC |
| 5 | RPL27 For | ATG GGC AAG AAG AAG ATC GC |
|  | RPL27 Rev | TCA AAC TTG ACC TTG GCC TC |
| 6 | RPL23 For | CTC TGG TGG CTG GAA TTG AC |
|  | RPL23 Rev | CCT TGC GTT TAA GAG CAG GA |
| 7 | RSL24D1 For | CGG AAA GCA GCT GGT AAA GA |
|  | RSL24D1 Rev | AGA GGG GCT CGG ATA AGA TG |
| 8 | LSM5 For | CTG CCC TTA GAG CTT GTG GA |
|  | LSM5 Rev | CTC CTC CAG GAA CCA GCA TTG |
| 9 | LRRN3 For | CAG CCA GAT TGC CAG CTA AC |
|  | LRRN3 Rev | AGT TGC TCA GTT CGG ACA GA |
| 10 | PDCD5 For | AGC CCA AGT TCT GGA TCA GT |
|  | PDCD5 Rev | TCG TCA TCT TCA TCA GAG TCC A |
| 11 | KRR1 For | GCC GGA GAA CCA AGA TGA AT |
|  | KRR1 Rev | GGT CCA GGG TTG CAT TAA CA |
| 12 | IL7 For | TCC CCT GAT CCT TGT TCT GT |
|  | IL7 Rev | AAT TGC CTC AAC TTG CGA GC |
| 13 | IL7R For | ACT GAC CTG TGC TTT TGA GG |
|  | IL7R Rev | CTC AGG TCA AAA GGA GCC TC |
| 14 | IL1 α For | CTG AAT GAC GCC CTC AAT CA |
|  | IL1 α Rev | CAG CAC TGG TTG GTC TTC AT |
| 15 | IL1β For | ATC CAG CTA CGA ATC TCC GA |
|  | IL1 β Rev | CGT GCA GTT CAG TGA TCG TA |
| 16 | PDCD1 For | CTG GGC GGT GCT ACA ACT |
|  | PDCD1 Rev | ACG AAG CTC TCC GAT GTG T |
| 17 | TNF-α For | TCC TAC CAG ACC AAG GTC AA |
|  | TNF-α Rev | TCG GCA AAG TCG AGA TAG TC |
| 18 | SOCS1 For | GAG AGC TTC GAC TGC CTC TT |
|  | SOCS1 Rev | GGG AAG GAG CTC AGG TAG TC |
| 19 | SOCS3 For | CAA GAC CTT CAG CTC CAA GA |
|  | SOCS3 Rev | CAG GTT CTT GGT CCC AGA CT |
| 20 | FOXP3 For | ACA GCA CAT TCC CAG AGT TC |
|  | FOXP3 Rev | CAC AAA GCA CTT GTG CAG AC |
